# Design of an Antibiotic-Releasing Polymer: Physicochemical Characterization and Drug Release Patterns

**DOI:** 10.3390/membranes13010102

**Published:** 2023-01-12

**Authors:** Himabindu Padinjarathil, Srikrishna Mudradi, Rajalakshmi Balasubramanian, Carmelo Drago, Sandro Dattilo, Nikhil K. Kothurkar, Prasanna Ramani

**Affiliations:** 1 Dhanvanthri Laboratory, Department of Sciences, Amrita School of Physical Sciences, Amrita Vishwa Vidyapeetham, Coimbatore 641112, India; 2 Department of Chemical Engineering and Materials Science, School of Engineering, Amrita Vishwa Vidyapeetham, Coimbatore 641112, India; 3 Institute of Biomolecular Chemistry, CNR, via Paolo Gaifami 18, 95126 Catania, Italy; 4 Institute for Polymer, Composite and Biomaterials, CNR, via Paolo Gaifami 18, 95126 Catania, Italy; 5 Center of Excellence in Advanced Materials & Green Technologies (CoE–AMGT), Amrita School of Engineering, Amrita Vishwa Vidyapeetham, Coimbatore 641112, India

**Keywords:** poly (ether ether ketone), sulfonation, physiochemical properties, ciprofloxacin, nalidixic acid sodium salt, drug release kinetics, mathematical model

## Abstract

Conventional drug delivery has its share of shortcomings, especially its rapid drug release with a relatively short duration of therapeutic drug concentrations, even in topical applications. Prolonged drug release can be effectively achieved by modifying the carrier in a drug delivery system. Among the several candidates for carriers studied over the years, poly (ether ether ketone), a biocompatible thermoplastic, was chosen as a suitable carrier. Its inherent hydrophobicity was overcome by controlled sulfonation, which introduced polar sulfonate groups onto the polymer backbone. Optimization of the sulfonation process was completed by the variation of the duration, temperature of the sulfonation, and concentration of sulfuric acid. The sulfonation was confirmed by EDS and the degree of sulfonation was determined by an NMR analysis (61.6% and 98.9%). Various physical properties such as morphology, mechanical strength, and thermal stability were studied using scanning electron microscopy, tensile testing, and thermogravimetric analysis. Cytotoxicity tests were performed on the SPEEK samples to study the variation in biocompatibility against a Vero cell line. The drug release kinetics of ciprofloxacin (CP) and nalidixic acid sodium salt (NA)-loaded membranes were studied in deionized water as well as SBF and compared against the absorbance of standardized solutions of the drug. The data were then used to determine the diffusion, distribution, and permeability coefficients. Various mathematical models were used to fit the obtained data to establish the order and mechanism of drug release. Studies revealed that drug release occurs by diffusion and follows zero-order kinetics.

## 1. Introduction

As the largest organ in the human body, the skin is the most susceptible to external trauma and injuries. These include burns, cuts, and other cutaneous injuries, which often lead to infections if not properly treated. The skin possesses marvelous regenerative abilities involving a very complex and heavily choreographed cascade of physiochemical processes. In diseases such as diabetes, this cascade is disturbed, owing to other physiological processes interfering with the regeneration of skin, and by putting the person at risk. Hence, wound management has been at the forefront of health research in recent times [1]. Studies in Europe revealed that between 27% and 50% of hospital beds are occupied by a patient requiring wound management [2]. About 1.5 to 2 million people live with a chronic wound across Europe. In the USA, an estimated 6.5 million people are afflicted with wounds at any time of year. Generally, wound-site drug delivery systems are cost-effective to manufacture [3]. The major hurdles in wound dressing are prolonged healing time, the need for frequent dressing changes, wound exposure, and the risk of infection [4]. In ancient times, certain leaves and cloth were used as dressing materials, along with plant-based extracts as ointments to prevent infection, reduce pain, and promote healing [5]. Delivering therapeutic agents through the dressings is relevant even today and there is a need to increase the efficacy and action of the agents. Another important factor that needs addressing is the maintenance of a therapeutic concentration of a drug over an extended period of time, as opposed to only short time after applying the dressing [6,7]. Sustained drug delivery is significantly more favorable compared to conventional topical application [3]. This can be achieved by modifying the material, physically or chemically, so that sustained release is achieved, and therapeutic drug concentrations are maintained in the wound vicinity, promoting favorable therapeutic outcomes. This requires the development of biocompatible polymers and polymer-based composites with properties such as degradation rates, drug diffusivity, and drug release profiles that are tunable by functionalizing the polymer [4].

Poly (ether ether ketone) (PEEK) is a linear, aromatic semi-crystalline polymer. It is one of the highest performing thermoplastics and has been reported to be biocompatible [8]. Another class of materials belonging to the same family is poly (arylene ether sul-phones) (PAES). Poly (arylene ether sulfones) are a class of membrane-forming engineering polymers renowned for their thermal and hydrolytic stability. Poly sulfone (PSF), poly (phenylene ether sulfone) (PES), poly (ether sulphone) (PES), poly (ether ether ketone sulfone) (PEEKS), poly (phenylene ether ether sulfone) (PEES), and poly (phenylene sulfone ether ketone) (PSEK) are some polymeric materials that are a subset of PAES. Poly (1, 4-phenylene ether ether sulfone) (PEES) (also called poly ether ether sulfone), is a polymer belonging to the same family and it also exhibits excellent film-forming properties. Because of this, it has been used as a membrane in fuel cells and catheters [5,8]. Among the several synthetic polymers used in drug delivery application, PEEK has been proven to control the release of therapeutics and it has an extremely versatile nature [9]. However, it has low moisture absorption and low permeability to fluids [10]. Nobuhiro Shibuya and Roger S. Porter proposed sulfonation as a method to increase the hydrophilicity of PEEK. It was observed that the degree of sulfonation is proportional to the aromatic ring concentration of the PEEK polymer [5]. Sulfonation of PEEK leads to increased swelling in water along with other changes in its properties. By controlling the extent of the swelling of sulfonated polyether ether ketone (SPEEK), it is possible to control the drug’s release rate. Nalidixic acid sodium salt (NA) is a synthetic antimicrobial/antibiotic agent used for research and is soluble in water. Ciprofloxacin (CP) is a quinolone antibiotic used to treat various bacterial infections since it arrests bacterial growth. These drugs are adequately stable towards temperature differences and allow easy handling [11].

The current work mainly focuses on SPEEK with different degrees of sulfonation as the matrix to achieve controlled drug release. The degree of sulfonation in SPEEK was controlled by varying the sulfonation time with concentrated sulfuric acid. The release rate of NA and CP from the samples with different degrees of sulfonation was measured. The current research is the first report describing the control of the delivery rates of nalidixic acid sodium salt and ciprofloxacin from SPEEK membranes by varying the degree of sulfonation.

## 2. Materials and Methods

### 2.1. Materials

PEEK (CAS No: 29658-26-2) (molecular weight 20,800 Da) was purchased from Gharda Chemicals (Mumbai, India). Nalidixic acid sodium salt (CAS No: 3374-05-8), ciprofloxacin (CAS No: 85721-33-1), and sulfuric acid (98%) were purchased from Sigma Aldrich (Mumbai, India). The solvents *N*, *N*-dimethylformamide (DMF), dimethylsulfoxide (DMSO), and *N*-methyl pyrrolidone (NMP) were obtained from Avra Synthesis Pvt. Ltd., (Hyderabad, India), Nice Chemicals Pvt. Ltd., (Eranakulam, India) and SRL Chemicals Pvt. Ltd., (Mumbai, India) respectively. All chemicals were used as received. Bruker Quantax 200 X-Ray spectrophotometer (Berlin, Germany), Bruker Avance 400 MHz NMR spectrophotometer (Bruker Italia, Milan, Italy), Bruker VERTEX70 spectrometer (RockSolid™ design, Mumbai, India), TA Instruments Q100 differential scanning calorimeter (SDT Q600/Q20,TA instrument, New Castle, DE, USA), Carl Zeiss RA-ZEI-001 (Gemini 300), Shimadzu UV-1900, SHIMADZU, Kyoto, Japan, Bio-Rad 550 Cat log no: 170-6750 (Bio-Rad, Tokyo, Japan).

### 2.2. Sulfonation of PEEK and Membrane Casting

**Figure 1:**
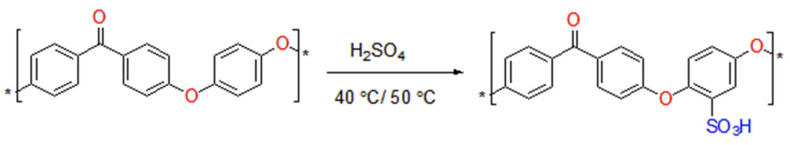
Sulfonation of PEEK.

The polymer was sulfonated by dissolving one part of the polymer in fifteen parts of sulfuric acid and heated to 40 °C and 50 °C for 1.5 h and 8 h, respectively. Sulfuric acid was chosen as a sulfonating agent for the sulfonation experiments, chiefly due to its relative ease of handling and affordability [12]. The sulfonation was carried out as per the following scheme in Figure 1.

At the end of the duration of sulfonation, the reaction mixture was quenched in ice-cold water to obtain sulfonated PEEK (SPEEK). The solid product was continuously rinsed with distilled water to remove the trapped sulfuric acid in the product. The white stringy form of SPEEK obtained after quenching was dried at room temperature and then at 60 °C in a vacuum oven to remove moisture. A portion of the dried polymer sample (100 mg) was dissolved in DMF (10–15 mL) for casting into a membrane. The solutions were cast into membranes by pouring them out into petri plates (2 inches diameter) and dried at room temperature for one day and in a vacuum oven at 60 °C for 4–6 h. Nalidixic acid sodium salt and ciprofloxacin were loaded using the following protocol, and the drug-to-polymer ratio was maintained at 1:10 *w*/*w* [10,13]. The specified amount of the drug (15 mg) was dissolved in DMF first, followed by the polymer dissolution in the drug solution. This yielded a viscous solution of the polymer and the drug in DMF, which was then poured out into a petri dish of a diameter of 2 inches, and dried at 60 °C under reduced pressure for about 8 h.

### 2.3. Degree of Sulfonation (DS)

#### 2.3.1. Confirmation of Polymer Sulfonation

The amount of sulfur in the SPEEK sample was estimated using EDS analysis on a Bruker Quantax 200 X-Ray spectrophotometer.

#### 2.3.2. Determination of the Degree of Sulfonation

The degree of sulfonation was calculated assuming all the sulfur to be present in the form of substituted sulfonic acid groups onto the polymer backbone. This analysis was performed using a Bruker Avance 400 MHz NMR spectrophotometer. This method was selected for the ease of determination of the degree of sulfonation compared with other previously reported methods. The sulfonation number and degree of sulfonation were calculated from the area under the NMR peaks of the SPEEK samples as per the formula given [12].

(1) $$\frac{n}{12-2n}=\frac{{\mathrm{AH}}_{E}}{\sum {\mathrm{AH}}_{x}}$$

where n is the sulfonation number, which when multiplied by 100 yields the degree of sulfonation.

AH_E_ is the area under the peak of the hydrogen on the sulfonic acid group.

ΣAH_x_ is the cumulative area of all the other peaks present in the spectra of the sulfonated polymer sample.

### 2.4. Membrane Characterization

The presence of sulfonic acid groups was verified using FT-IR (Bruker VERTEX70 spectrometer). Thermogravimetric analysis (TGA) of the sulfonated polymer samples was conducted on a TA Instruments SDT Q600 to assess the stability and changes in the onset of decomposition post-sulfonation. The glass transition temperature (Tg) was measured using a TA Instruments Q100 differential scanning calorimeter. The morphology of the SPEEK membranes was studied using a field-emission scanning electron microscope (Carl Zeiss RA-ZEI-001). The thickness of the solution-cast films of the four samples was measured using a surface profilometer. Tensile testing of the SPEEK membranes with different degrees of sulfonation was carried out on Dak System Inc.’s universal testing machine at room temperature using a 500 kg load cell at 5 mm min^−1^. Tests were carried out on five membrane samples at two degrees of sulfonation.

### 2.5. Solubility and Water Uptake Studies

The polymer’s solubility was checked in DMF, DMSO, and NMP to determine a suitable solvent for membrane casting [14,15]. Water uptake studies were performed by immersing the polymer membrane in deionized water and simulated body fluid (SBF) for about 8 h. The dry and wet membrane weights were recorded until a constant hydrated weight was observed. These values were used to calculate the membrane’s water uptake capacity using the formula reported in the literature [16].

(2) $$\mathrm{Water}\;\mathrm{Uptake}=\left[\frac{W_{wet}-W_{dry}}{W_{dry}}\right]\times 100\;\;$$

### 2.6. Physicochemical Studies

#### 2.6.1. Distribution, Diffusion, and Permeability Coefficients

The drug concentration was estimated using UV-visible spectroscopy (Shimadzu UV-1900; 200–600 nm). The estimation was performed using a correlation curve between the drug concentration and the absorption at 331 nm for nalidixic acid sodium salt and 313 nm [12,14,17] for ciprofloxacin. The diffusion coefficient (*D*) was calculated based on Fick’s first law [16]:

(3) $$D=\frac{h^{2}}{6tL}$$

where ‘*h*’ is the thickness, and ‘*tL*’ is the lag time acquired from the graph plotted of time vs. concentration of the drug within the system. The distribution coefficient (*K*), which is a measure of the concentration of the drug in the solution, was calculated from the slope of time vs. concentration plot. The permeability coefficient (*P*) is the transport flux of the drug through the membrane per unit of membrane thickness. It is a function of the distribution coefficient (*K*) and the diffusion coefficient (*D*) [18,19] as given by the following expression:

(4) $$P=\frac{KD}{\Delta x}$$

#### 2.6.2. Drug Release Kinetics

The drug release kinetics were studied by absorption studies (UV-vis spectroscopy). A drug-loaded sulfonated polymer membrane of diameter 2 inches and a thickness of 60 µm was immersed in 100 mL of deionized water (pH7) and stimulated body fluid (SBF) with periodic agitation [10,11]. About 3.5 mL of the solution was withdrawn at different time intervals and subjected to UV-vis absorption spectroscopy measurements, with deionized water containing a polymer membrane without a drug as the blank, and a path length of 10 mm. All plots and graphs used the absorbances at 331 nm (NA) and 313 nm (CP) [5]. The quantity of drug released into the solution was calculated by correlating the absorbance at different time intervals with the absorbance of standard solutions of the drug. The distribution coefficient of both the drugs in the polymer/water system was calculated from these values. 

### 2.7. In Vitro Biocompatibility Studies

The SPEEK membrane was sterilized in an autoclave at 15 °C for 15 min. Solutions were prepared from 32 mg/mL stock solutions in DMSO and diluted to the required concentration using DMEM plain media for the treatment process. Vero cell line cells (epithelial cells obtained from the African Green Monkey) were cultured (5 × 10^4^ cells per well), seeded on 96-well plates in Dulbecco’s modified Eagle’s medium (DMEM, Sigma, St. Louis, MO, United States) with 10% FBS, and incubated at 37 °C. After 24 h, the cells in the well became confluent and were treated with different stock solution concentrations. After incubation at 37 °C for 24 h, the medium was replaced with a fresh medium (1 mL), and MTT (6 mg/10 mL of MTT in PBS) was supplied to each well. This was again incubated at 37 °C for 4 h. Following this, the supernatant was slowly drained, and 1 mL DMSO was added to each well. The absorbance of the solution was estimated using a microplate reader (Bio-Rad 550) at 570 nm to ascertain the optical density (OD) value. The cell viability was calculated by an assay using the following expression:

(5) $$\%\;\mathrm{Inhibition}=\left[100-\left(\frac{\mathrm{OD}\;\mathrm{of}\;\mathrm{Sample}}{\mathrm{OD}\;\mathrm{of}\;\mathrm{Control}}\right)\right]\times 100$$

### 2.8. Statistical Analysis

The results are expressed as mean ± standard deviation (SD) of three replicates. Statistical significance (5%) was evaluated by one-way analysis of variance (ANOVA) followed by the Student’s *t*-test *p* < 0.05. All statistical analysis was performed using OriginPro 2018 software (OriginLab Corporation, Northampton, MA USA).

## 3. Results and Discussion

PEEK is a polymer that has found applications in implants such as dental implants, orthopedic implants, and much more, owing to its biocompatibility and the fact that the polymer is inert to chemical changes, even in a biological system. The polymer has poor solubility in polar solvents, and it can be finely tuned by introducing hydrophilic groups into the backbone. A simple and well-known technique is the electrophilic substitution reaction by sulfuric acid. Among several sulfonating agents, sulfuric acid is one of the better choices due to economic constraints and the relative ease of handling. According to R. Y. M. Huang et al., and S. Shanmuga et al. [10,20], sulfonation is a convenient method to improve the solubility of PEEK in commonly used solvents. It was also found that the solubility of sulfonated PEEK varied with the degree of sulfonation. A comparative study was carried out between two samples, with one sample being sulfonated for 1.5 h at a temperature of 40 °C (SA-01) and the other being sulfonated for 1.5 h at a temperature of 50 °C (SA-02). The characterization, qualitative and quantitative, of the modified polymer is paramount since the properties can change drastically during the sulfonation, and documentation of these properties for further studies is essential in a comparative study of the drug release kinetics and other properties of the samples.

### 3.1. Degree of Sulfonation of SPEEK

#### 3.1.1. Reaction Optimization and Confirmation of Sulfonation

The degree of sulfonation is the percentage of monomers in the polymer chain that have undergone a successful electrophilic substitution reaction, with the sulfonate groups as the attacking group. The reaction conditions were optimized by a trial-and-error method, and the sulfonated products were compared by their dissolution times in deionized water. The dissolution time of the sulfonated product varied positively with the degree of sulfonation. Initially, two different temperatures and times of reaction were taken. The yield of sulfonated product decreased with an increase in sulfonation as seen in Table 1. This proves that higher degrees of sulfonation leads to some degradation of the polymer backbone. However, the suitability of the SPEEK samples for the drug delivery application can be more intuitively assessed based on their dissolution time in water. Hence, Table 1 describes the dissolution times of SPEEK sulfonated under different conditions of temperature and reaction time.

Both the temperature of sulfonation and the reaction time influenced the dissolution time of the sulfonated product. However, since shorter reaction times at a higher temperature are more convenient and potentially more economical than long reaction times at a lower temperature, temperature was used to control the degree of sulfonation. The reaction time was fixed at 1.5 h. Apart from the samples listed in Table 1, samples were also sulfonated at 80 °C. However, in these cases, the product solubility was too high and only a small quantity could be recovered by filtration and drying. Hence, samples of the polymer sulfonated at 80 °C were excluded from all subsequent studies.

#### 3.1.2. EDS Analysis to Confirm the Sulfonation of the Polymer

The sulfur content of the sulfonated samples was estimated by an elemental analysis using the EDS technique. The atomic percentages of carbon, hydrogen, nitrogen, and sulfur within the samples are detailed in Table 2.

As expected, sulfur was absent in the neat PEEK sample, while it was present in the SPEEK samples SA-01 and SA-02. The increase in the sulfur content from 3.88% (SA-01) to 14.5% (SA-02) clearly indicates that an increase in reaction temperature from 40 °C to 50 °C increased the degree of sulfonation of the product for the same reaction time of 1.5 h.

#### 3.1.3. Determination of the Degree of Sulfonation by NMR Spectroscopy

**Figure 2:**
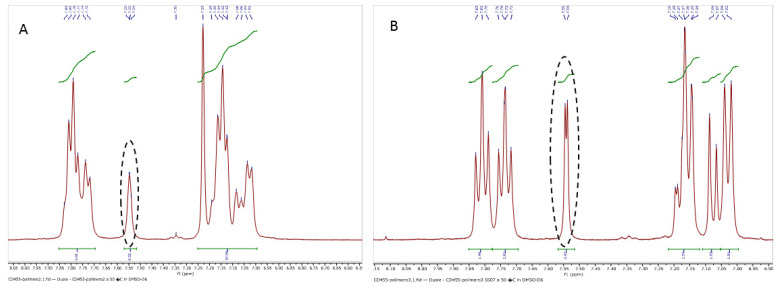
NMR spectra of sulfonated PEEK samples (**A**) SA-01 (degree of sulfonation: 61.6%) and (**B**) SA-02 (degree of sulfonation: 98.9%).

NMR spectrophotometric analysis was used to confirm the sulfonation of the PEEK and to quantify the number of sulfonic acid groups substituted onto the polymer chain (degree of sulfonation). NMR studies were carried out in deuterated DMSO. The degree of sulfonation was calculated from the 7.5 and 7.6 ppm peaks as in Figure 2. The ratio of the area under this peak to the sum of the areas of all other peaks is related to the sulfonation number (n) and the degree of sulfonation (DoS) as described in the Materials and Methods section. Accordingly, the DoS were calculated to be 61.6% for the sample SA-01 and 98.9% for SA-02.

### 3.2. Casting of the SPEEK Membrane and Its Characterization

Huang et al. found SPEEK to be soluble in various solvents [20]. Three solvents, namely, DMSO, DMF, and NMP, were shortlisted for the casting of membranes of the SPEEK samples. DMSO had the advantage of also being the solvent used in the cytotoxicity studies; however, it was eliminated due to the sparing solubility of the polymer in it. DMF and NMP have been used as solvents for sulfonated PEEK in several reports [21]. However, NMP was not a very good solvent of SPEEK and required sonication, whereas DMF was found to be a good solvent. Besides, DMF has a lower boiling point as compared to NMP [10,13]. That, along with the lack of necessity for sonication for dissolving the polymer, makes a DMF-based solution casting process of SPEEK more energy efficient compared to an NMP-based process. Hence, DMF was used for the solution casting of all membranes in this study. Both the drugs used in the study were found to be soluble in DMF.

#### 3.2.1. Mechanical Characterization

The membrane’s tensile strength was found to be directly proportional to the DoS [22,23]. The SPEEK membrane exhibited Young’s moduli of 32.9 and 88.1 N/sq.mm, respectively, for the higher and low sulfonated polymer samples. The elongation factor of the lower sulfonated membrane (SA-01) was higher when compared with SA-02 (Table 3).

The mechanical properties are important because when this drug-loaded polymer is used as part of a wound dressing, it can be subjected to tensile forces causing the membrane to rupture. The Young’s modulus was found to decrease with an increase in the DoS of the polymer [22]. Reyna Valencia and S. Kaliaguine performed a study to evaluate the tensile and mechanical properties of SPEEK and BPO4/SPEEK composite membranes [23]. The tensile strength of the polymer membranes changed with the DoS. It was proposed that the sulfonation of the PEEK polymer reduced the brittleness of the polymer. PEEK specimens exhibited brittle behavior, whereas SPEEK films displayed ductile behavior [22].

The thickness of the membranes was measured by surface profilometry, and it was observed that the membrane thickness was quite uniform within 60–70 µm and the diameter was about 0.025 m, which was the diameter of the petri dish in which they were cast. The uniformity in the thickness was maintained by adding the same amount and concentration of the polymer solution to identical petri dishes.

#### 3.2.2. FT-IR Spectra of the SPEEK Membrane

**Figure 3:**
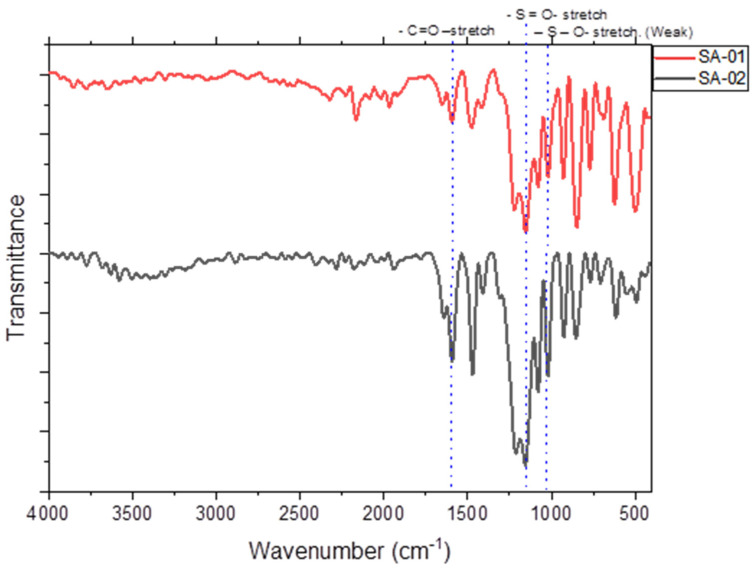
FTIR spectra of sulfonated PEEK samples, SA-01 (red), and SA-02 (black).

The chemical characterization of the samples was carried out using Fourier transform infrared spectroscopy (FT-IR) in the ATR mode. The IR transmittance was found to reduce with increasing sulfonation, which has also been reported in the literature [24,25,26]. The aromatic C-H stretching peaks were observed in the range of 3636 and 3500 cm^−1^ in both SA-01, and SA-02, respectively. While the backbone carbonyl peak at 1647 cm^−1^ remained unchanged in SA-02, a slight peak was observed in SA-01 around that region. A sharp asymmetric stretching peak of the SO_2_ bond was observed in SA-02 at 1316 cm^−1^, whereas a wide absorbance was observed in SA-01 in the range 2000–500 cm^−1^. The asymmetric and symmetric stretching of SO_2_ around 1069 cm^−1^ and 1153 cm^−1^, respectively, were observed in SA-02. In the case of SA-01, the same peaks were broadened and were of a lower intensity than that of SA-02. The C=O asymmetric stretching peak was seen in the 2200–2100 cm^−1^ range in both SA-01 and SA-02 (Figure 3), since, the relative intensity of absorbance peak dependence on the molar amount of SO_2_ moieties present. SA-01, with lower DoS, exhibited lower intensity peaks compared to SA-02 at 1153 cm^−1^.

The primary frequencies that were looked up were for the sulfonate functional group (sulfur–oxygen double bond and sulfur–oxygen single bonds), which could be observed in both cases [24]. The observed higher intensity with the higher DoS sample, SA-02 as compared to SA-01, was as expected. According to Weigeng Wang, SPEEK has a characteristic peak of 1067 cm^−1^ to 1650 cm^−1^. Increased reaction time leads to a greater degree of sulfonation due to the incorporation of more polar sulfonic groups [25]. The peak intensity increased with the degree of sulfonation, and the peaks became broader. They even suggested that the carbonyl backbone of the polymer remained unchanged. According to Marius Behnecke, the presence of a symmetric O=S=O band in the region confirms sulfonation. The peaks were due to the residual number of sulfonated links with peaks at 1480 cm^−1^, indicating a modification in aromatic rings. Peaks at 630 cm^−1^ meant that the bonds between carbon and sulfur could be confirmed via the bands corresponding to C-S vibrations [26]

#### 3.2.3. Water Uptake Studies

Reyna Valencia and S. Kaliaguine performed a water absorption test using SPEEK films, correlating the water absorption with the degree of sulfonation [23]. In this study, we performed water uptake studies by immersing the SPEEK membrane in deionized water for about 8 h and weighing it. Post-weighing, the membrane was subject to drying, and the dry weight was recorded. This process was repeated until a constant weight was observed. The sample with the higher degree of sulfonation (SA-02) was observed to absorb 67.69% of water by weight at the highest measurement. It was further observed that this polymer membrane could hold approximately 2.1 times more water than its actual weight. The polymer with a lower degree of sulfonation (SA-01) was observed to measure up to 51.12% of water by weight at the highest measurement. It was observed that this polymer membrane could hold approximately 1.05 times its weight in water.

The introduction of the sulfonate groups as a hydrophilic substituent in the polymer backbone increases the hydrophilicity of PEEK, enabling the polymer to hold water. The extent to which the membrane becomes hydrophilic influences the mode of drug release, i.e., erosion or diffusion. If the membrane becomes too hydrophilic, it will tend to dissolve in water, in which case the drug release would mainly be due to the erosion of the membrane compromising its structural integrity. A lower hydrophilicity, corresponding to a lower uptake of water would make Fickian diffusion the mode of drug release since the structural integrity of the membrane would not be compromised. The drug release mechanism in this study was confirmed to be diffusion after applying different mathematical models [27,28] to the data.

#### 3.2.4. Thermogravimetric Analysis of the Membrane

The thermogravimetric analysis was carried out with a shallow platinum crucible as the sample holder in a nitrogen atmosphere. The first observed weight loss was attributed to the loss of water used in the preparation of the films, which might have been trapped in the polymer matrix.

**Figure 4:**
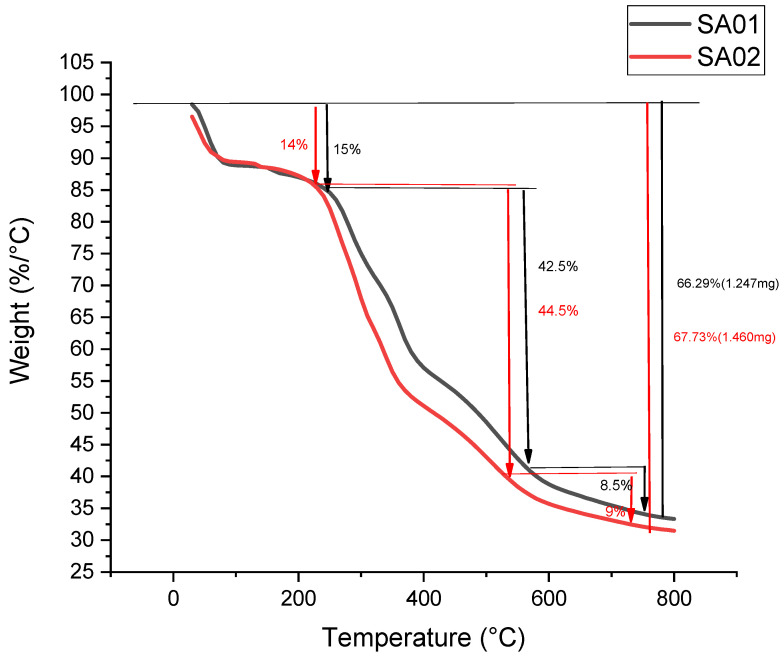
Thermogram of PEEK; SA-01 and SA-02.

The subsequent loss of weight, which was more significant, was due to the ejection of sulfur trioxide from the polymer matrix by the decomposition of the sulfonate groups introduced into the polymer matrix, as shown in Figure 4. The following weight loss can be correlated to the decomposition of the polymer chain. Some loss in the weight of the sample was observed, although these were minimal changes since the significant loss in weight of the sample was observed after the temperature broke the barrier of 580 °C, the temperature at which the analysis was capped in this case. From the article reported by Patel et al., the decomposition product at 450 °C can be attributed to the decomposition of the polymer chain into 4-phenoxy phenol and 1,4-diphenoxybenzene, and the steady reduction until 650 °C resulted in the formation of carbon monoxide and carbon dioxide, which was then arrested, utilizing the stoppage of the analysis in our case [29].

#### 3.2.5. DSC Analysis of Membrane

The differential scanning calorimetry (DSC) method was used for the determination of the glass transition temperature (Tg) for SPEEK membranes SA-01 and SA-02.

**Figure 5:**
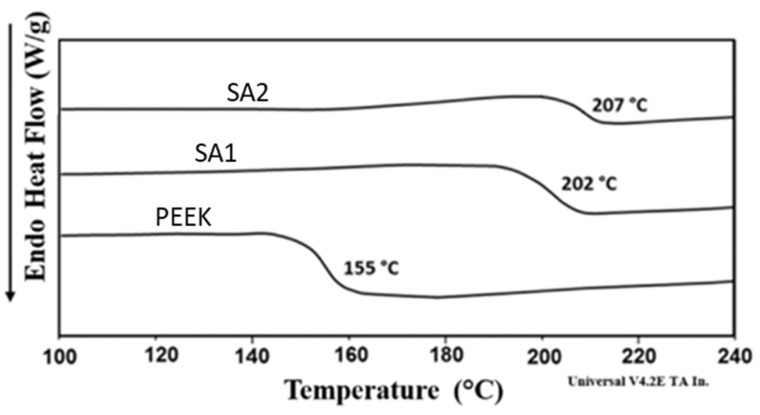
DSC curve of PEEK; SA-01 and SA-02.

An increase in the glass transition temperature was recorded in the sulfonated polymer samples in comparison with the unsulfonated PEEK sample, as shown in Figure 5. This can be attributed to the strong interactions between the sulfonate groups within the polymer chain which increased with DoS. These interactions were responsible for hindering segmental rotations in the polymer chain, resulting in an increase in the glass transition temperature (Tg) with increasing DoS.

#### 3.2.6. SEM Images of the Drug-Loaded SPEEK Membranes

Previous reports about polymers used as carriers for drug release have reported that sustained release is best achieved when the drug is observed to form a thin layer on the surface of the polymer membrane, which was relatively easily achieved in this case [1,22].

**Figure 6:**
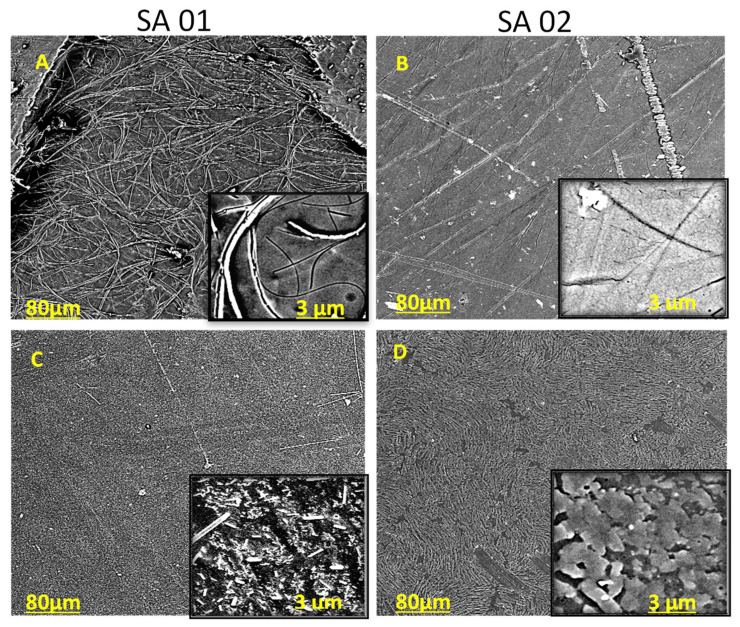
(**A**–**D**): Surface Images of (**A**) SPEEK SA-01 film of DS = 61.6% loaded with nalidixic acid sodium salt, (**B**) SPEEK SA-02 film of DS = 98.9% loaded with nalidixic acid salt, (**C**) SPEEK film (SA-01) with ciprofloxacin and (**D**) SPEEK film (SA-02) with ciprofloxacin SEM image at magnification 40.0 K X and 200 nm.

The SEM images of the SPEEK membranes with NA in SA-01 and SA-02 (Figure 6A,B) and ciprofloxacin (Figure 6C,D), respectively. As expected, the neat SPEEK film showed prominent features of SA-01, and non-uniform layers were seen in SA-02. A rod-like domain structure was observed in SA-01 (Figure 6A) loaded with NA, whereas in the ciprofloxacin-loaded counterpart, more of the drug was loaded compared with NA. The drug-loaded SA-02 film (Figure 6B,D) showed a phase-segregated morphology coating layered over circular domains corresponding to the drug-rich phase, which became prominent as the rate of sulfonation was increased in the case of both drugs. The drug embedded in the membrane of the SA-02 film (Figure 6B,D) showed a non-homogenous coating to form a layer over the surface of the polymer compared with that of SA-01, which was irregular and granulated. This shows that the increased sulfonation of the polymer enhanced its capacity to hold drug molecules.

### 3.3. Physicochemical Studies of the Drug-Loaded SPEEK Membrane

#### 3.3.1. Distribution, Diffusion, and Permeability Coefficients

The distribution coefficient of the drug molecules in the water–polymer membrane system was calculated by employing the amount of drug released at the end of 8 h and the total amount of drug loaded into the membrane. The distribution coefficients of nalidixic acid sodium salt and ciprofloxacin in the polymer with a higher degree of sulfonation (SA-02) in a water system were determined. It was observed that the distribution coefficients were 0.24 and 0.30, respectively. The distribution coefficients of the drug molecules in the lower sulfonated polymer in the water system were 0.2968 for nalidixic acid sodium salt and 0.2302 for ciprofloxacin.

The diffusion coefficient of the water–polymer system was determined by Fick’s Law of Diffusion [28]. The diffusion coefficient of the polymer membrane with a higher degree of sulfonation (SA-02) was observed to be 1.2683 × 10^−9^ cm^2^/s, and the lower sulfonated polymer membrane SA-01 had a diffusion coefficient of 1.5174 × 10^−9^ cm^2^/s.

The permeability coefficient was calculated from the distribution and diffusion coefficients equation which is governed by Overton’s Rule [10]. At first, the permeability coefficient of the nalidixic acid sodium salt-loaded membrane (SA-02) was observed to be 5.9829 × 10^−6^ cm/s, while that of the ciprofloxacin-loaded membrane was observed to be 7.5697 × 10^−6^ cm/s. The lower sulfonated SA-01 showed a permeability of 6.5752 × 10^−6^ cm/s for the nalidixic acid sodium salt-loaded membrane and 5.4430 × 10^−6^ cm/s for the ciprofloxacin-loaded membrane (Table 4). These properties are based on the polymer matrix material, which has a crucial role in diffusion, permeation rate, etc. Earlier studies of SPEEK with different DoS and drugs showed similar patterns of diffusion and permeability coefficients of 1.36 × 10^−5^ cm/s and 0.029–0.203 cm^2^/ h [18]. Moreover, the crossing linking agent between the polymer and the active material and their concentration is another factor that influences these values [30].

#### 3.3.2. Drug Release Kinetics

**Figure 7:**
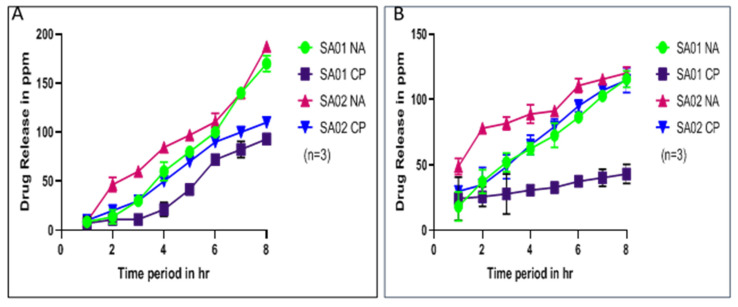
Drug release kinetics of SPEEK membranes SA-01 and SA-02 with two drugs in (**A**): simulated body fluid (SBF) and (**B**) deionized water in both nalidixic acid sodium salt (NA) and ciprofloxacin (CP). Lines indicate drug release concentration in ppm for 8 h.

The drug release kinetics were investigated by absorption studies using UV spectroscopy (Shimadzu UV—1900) [6,31,32]. The quantity of drug released was correlated with the absorbance values of standard solutions of the drug in simulated body fluid and water with three repetitions to obtain an average release (Figure 7). The quantity of drug released was evaluated by determining the absorbance of standard solutions of the drug in water and by correlating the absorbance of the solutions in the release experiments. Graphs depicting absorbance vs. time and concentration of release in ppm vs. time were plotted for further calculations [33]. The release of nalidixic acid sodium salt in this case agreed with the solubility constraints of the drug, as by the end of the study, it was estimated that the amount of drug present in the SBF system was about 10 mg per trial (Supporting Information Figures S2 and S3). Since the amount of SBF solution used was 100 mL for every trial, this suggests the study was carried out within the confines of the solubility of NA.

These coefficients were predominantly employed to determine the polymer’s ability to be a suitable carrier. The distribution coefficient determined the distribution of the active ingredient loaded into the membrane between the water and the organic “solvent” system, in this case, water, and the sulfonated polymer film. This coefficient can be used to estimate the quantity of the active ingredient in the carrier with specificity to the time intervals.

The diffusion coefficient relates the concentration gradient of the drug in the system with its molar rate of diffusion. This value can be used to study the relative ease of the drug molecule to diffuse from one system into the other. It is said that a higher diffusion coefficient endorses a faster diffusion of the drug from one system to the other. This coefficient can be used to define the instantaneous rate of dissolution of the drug from the carrier system. The permeability coefficient is determined as a quantitative relation that defines the rate of the molecule’s ability to cross a membrane. This value defines the rate of the whole drug release study and can also be used to understand the mode of drug release, i.e., if the drug release is by the diffusion mechanism or the swelling mechanism. This can be determined by a direct linear relation. The higher the coefficient, the higher the possibility of the release following a swelling mechanism since a higher coefficient means a higher amount of drug released by the system. This scenario is possible in the case of an uncontrolled water uptake, which can be termed swelling.

Figure 7 illustrates the results of performing the drug release kinetics experiment in deionized water and stimulated body fluid (SBF). The experiments were performed for over 8 h. It can be observed that sustained drug release was achieved with the polymer on sulfonation. It can also be observed that the drug release in the case of ciprofloxacin was similar in both the polymers, but in the case of nalidixic acid sodium salt, the drug release pattern was better in the case of the polymer with a higher degree of sulfonation since the amount of drug released in the case of the polymer with the lower sulfonation was higher at a lower time. It then increased slightly compared to the other case, where the drug release was more uniform over 8 h. The result was correlated with the absorbance of standard solutions of the drug in water and a similar pattern was observed in SBF. These values were further used to fit the drug release into various drug release kinetics models.

### 3.4. Mathematical Models of Drug Release

The drug release pattern determined by experimental means was verified using four significant mathematical models: zero-order kinetics, first-order kinetics, Hopfenberg, and Ritger–Peppas models [33]. The experimentally obtained values of the parameters involved in drug release were substituted in the model of interest to arrive at results that conformed to the expected trend. This study involved the employment of polymers with two different degrees of sulfonation, and two active ingredients (ciprofloxacin and nalidixic acid sodium salt), yielding four possible combinations, all of which were used in this study. Zero-Order Kinetics is governed by the model, with the assumption of the consumption of a reactant over time, forming a product (Tables S1–S4). It follows a trend similar to the y = mx plot, where the plot’s slope is the reaction rate. The equation that governs this model is 𝑓_1_ = 𝑘_0_, where f_1_ is the fraction of the active agent released into the system (𝑓_1_ = 1 − (*W_I_*/*W*_0_)) at time *t* and *k*_0_ is the release velocity constant. Although this model can be applied for the calculations and verification to check if the drug release follows zero-order kinetics, from the figures represented above, it can be seen that over time, the release of the active agent was independent of the concentration of the agent in the matrix, which is a common notion in a zero-order reaction, which is true [27]. In this case, the drug release was almost linear, falling in line with a zero-order release pattern. So, it can be assumed that the drug release followed zero-order kinetics.

First-order kinetics is explained using this formula log*Q*_1_ = log*Q*_0_ + *k*_1_ ∗ *t*/2.303 where, *Q*_1_ is the total amount of drug released at time *t*, *Q*_0_ is the total amount of drug in the system/carrier, and k_1_ is the rate constant for the process. A graph depicting time vs. quantity was plotted considering the equation y = mx + c, which corresponds to the equation of a straight line (Supplementary Data). The values obtained after calculation showed a steady decrease in the magnitude of the first-order rate constant (measured in s^−1^). This was observed for all three samples except SA-02 (the higher sulfonated PEEK with the nalidixic acid sodium salt drug), where there were slight deviations. In this case, the dependence of the release characteristics could not be correlated with the drug’s initial concentration within the polymer membrane matrix. Hence, it can be concluded that the drug release pattern did not obey first-order kinetics.

The Ritger–Peppas model is governed by the equation: $\frac{M_{t}}{M_{\infty }}=kt^{n}$, where *M_t_* is the amount of drug released at time *t*, *M*_∞_ is the amount of drug released from the system at equilibrium/total amount of drug-loaded into the carrier, *k* is the constant associated with release velocity, and *n* is the exponent of release, dictated by the geometry and the mode of drug release. The mathematical differences are observed only in variation in the exponent of the equation. The diffusion mechanism is governed by the Fickian model (*n* ≥ 0.5), whereas the non-Fickian model governs various anomalous cases (0.5 < *n* < 1) and belongs to the zero-order of release. The swelling and anomalous cases exclusively fall under the non-Fickian model [28].

The release of the drugs NA and CP in SA-01 and SA-02 (Figure S2) fell within the Fickian model, where the value of *n* was observed to be 0.5, which reconfirmed that it did fall in the zero-order of drug release. The Hopfenberg Model of Drug Release Kinetics was employed to confirm that the drug release occurred by diffusion and not erosion. The Hopfenberg Model is employed in the case where the driving force for the drug release is the erosion of the carrier.

The equation that dictates the drug release, in this case, is:

(6) $$\frac{M_{t}}{M_{\infty }}=1-{\left[1-\frac{k_{o}t}{C_{o}a_{o}}\right]}^{n}$$

where *M_t_* is the amount of drug released into the system at time *t*, *M*_∞_ is the amount of drug released at equilibrium state/total amount of drug-loaded into the carrier, *k_o_* is the erosion grade constant, *C_o_* is the concentration of drug in the matrix, *a_o_* is the half part thickness of the film (determined based on geometry), and n is the geometrical constant (determined based on geometry) [28].

This model was employed for two specific reasons. The first reason was to calculate any degradation that could not be observed in the polymer during the experiment. The other reason was to verify that the drug release was predominantly due to diffusion with significant erosion of the carrier. This can be established by the determination of the erosion constant in the equation. The value was small, so the erosion was proportionally small. In all cases, it was observed that the carrier’s erosion constant in the Hopfenberg Model was in the order of 10^−4^ m/h (Tables S1–S4). This can be attributed to the fact that degradation was insignificant in contributing to the drug release due to the diffusion mechanism.

### 3.5. In Vitro Cytotoxicity Studies of the SPEEK Membrane

**Figure 8:**
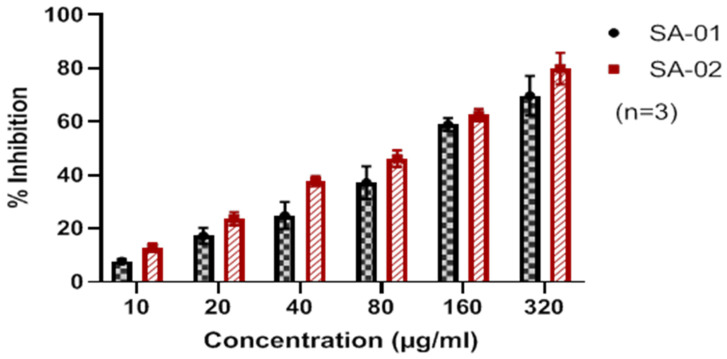
In vitro cytotoxicity studies of SA-01 and SA-02 in Vero cell lines (n = 3).

Evaluation of cytotoxicity is a crucial step in understanding the biocompatibility of a polymer membrane when it comes in contact with the biological system and also understanding other interactions which will ensure that the damage to healthy cells is minimum. Hence, the cytotoxicity was evaluated on a Vero Cell line by the MTT assay (Figure 8).

It was observed that there was a linear relationship between the degree of sulfonation and the cytotoxicity against a Vero cell line. The IC_50_ values of SA-01 and SA-02 were 118.90 ± 2.978 μg/mL and 98.34 ± 3.11 μg/mL, respectively. From above, even though the time interval of sulfonation varied, a similar degree of sulfonation showed a nearly identical IC_50_ value.

The biocompatibility of PEEK has been well documented and, over time, has also been a significant contender in the design of various biological replacements, including implants with dental and orthopedic applications [10]. It is necessary to evaluate the extent to which the sulfonation affects the biocompatibility of the polymer. The less sulfonated PEEK exhibited a higher biocompatibility with an IC_50_ value above 100 μg/mL. The work of Zhao and his colleagues explained that the sulfonation of PEEK and its subsequent immersion in water produced a 3D nano-structured network bearing bio-functional groups, enabling the preparation of two types of SPEEK samples [34,35]. The in vitro results demonstrated the ability of SPEEK-WA to induce pre-osteoblast functions such as initial cell adhesion, proliferation, and in vitro osteogenic differentiation, in addition to substantially enhanced osseointegration bone-implant bonding strength in vivo and apatite-forming ability [36]. The chief reason for this work’s cytotoxicity value above 100 μg/mL is attributed to the presence of residual sulfonic acid groups. Summarizing the results, the mathematical models successfully explained controlled zero-order kinetics by the diffusion mechanism and proved its biocompatibility.

## 4. Conclusions

PEEK was sulfonated under different conditions, and the polymers sulfonated by 61.6% and 98.9% were considered for further applications. The dissolution patterns of both the SPEEK membranes were studied in water and SBF, from which it was observed that the former underwent swelling while the latter underwent dissolution in SBF. The percentage of sulfur content in the sulfonated polymer was also verified by elemental analysis, and only a slight deviation from the value obtained in the titration method was noticed. The enhanced water-uptake properties of SPEEK, when compared to PEEK, were also observed to be higher for the polymer sulfonated by 98.9%, as it could hold nearly two times more water than its actual weight. A TGA analysis of both low and high sulfonated PEEK samples gave a lucid picture of their weight loss at subsequent stages. From the pharmacokinetic studies of the characterized drug-loaded polymers, it was concluded that ciprofloxacin exhibited sustained release from higher and lower sulfonated PEEK; on the other hand, nalidixic acid sodium salt showed the expected sustained release pattern only in the case of the higher sulfonated SPEEK. The nature of drug release was zero-order in all the situations, as predicted by the mathematical models discussed in Section 3.5. An evaluation of the cytotoxicity of the SPEEK membranes by Vero cell line gave nearly identical IC_50_ values for all the samples, thus proving their biologically benign nature. Two practical limitations include the initial burst in the drug release and the limited biocompatibility of the sulfonated polymer. These studies have shown that the drug release followed zero-order kinetics, and the diffusion mechanism governed the release mode. A thorough literature survey, which was undertaken before the study with this polymer in the biological space, substantiated that PEEK is an up-and-coming contender for employment as implants in dental and orthopedic scenarios, along with possible prosthetic applications. This study is an addition to this polymer’s already extensive biological applications. It can also be said that this might not limit the myriad other possibilities where the polymer could prove to be a worthy candidate.

## Figures and Tables

**Table 1:** Influence of sulfonation temperature and the time of reaction yield and the solubility of SPEEK samples.

Temperature (°C)	Time of Reaction (in Hours)	Yield (%)	Dissolution Time (in Hours)
40	1.5	73.33 ± 4.6	12
40	8	65.7 ± 3.5	4
50	1.5	59.2 ± 2.1	9
50	8	53.7 ± 1.4	3

**Table 2:** The elemental composition of PEEK and two samples of sulfonated PEEK with different degrees of sulfonation obtained from an EDS analysis.

Sample Name	C (%)	S (%)	H (%)	O (%)	Sample Weight (mg)
PEEK	78.69	0	1.53	19.79	7.96
SA-01	61.75	3.88	4.13	30.24	6.85
SA-02	45.64	14.50	9.02	33.75	8.08

**Table 3:** Tensile strengths and Young’s moduli of sulfonated PEEK samples with different degrees of sulfonation.

Samples	Young’s Modulus (N/sq.mm)	Elongation Factor (%)	Stress Yield (N/sq.mm)	Stain Yield (mm)
SA-01	88.1 ± 0.87	77	20.73 ± 0.09	62.88
SA-02	32.9 ± 1.21	17.44	4.9 ± 0.1	5.33

**Table 4:** Physicochemical properties of SPEEK.

Samples	DS (%)	Distribution Coefficient		Thickness (mm)	Permeability Coefficient (cm/s)	
		Nalidixic Acid Sodium Salt	Ciprofloxacin		Nalidixic Acid Sodium Salt	Ciprofloxacin
SA-01	61.6	0.2469 ± 0.07	0.3066 ± 0.05	0.088	6.5752 ± 1 × 10^−6^	5.4430 ± 3.1 × 10^−6^
SA-02	98.9	0.2968 ± 0.06	0.2302 ± 0.07	0.11	5.9829 ± 0.8 × 10^−6^	7.5697 ± 1.7 × 10^−6^

## Data Availability

All the data pertaining to the work are furnished in the manuscript.

## Supplementary Materials

The following supporting information can be downloaded at: https://www.mdpi.com/article/10.3390/membranes13010102/s1, Figure S1. (A) Graph illustration of linear equation of drug release from SPEEK membranes SA01 and SA02 with two different drug Nalidixic acid (NA) and ciproflaxcin (CF) to show first order kinetics. (B) Graph illustration the Drug release velocity (n) from SPEEK membrane SA01 and SA02 with (Nalidixic Acid and Ciproflaxcin) in 1 to 8 h period; Figure S2. Drug in various concentraions (A) Nalidixic acid (NA) and (B) ciproflaxcin (CF); Figure S3: Linear equation of drug release from SPEEK membranes. (A) SA01 and (B) SA02with Nalidixic acid (NA). (C) SA01 and (D) SA02 with Ciprofloxacin for 1 to 8 h period; Table S1. Data showing the First order, Zero order kinetic, Hopfenberg Model (Erosion Coefficient (m/h) and Ritger -Peppas model of SPEEK membrane SA01 with nalidixic acid sodium salt in 1 to 8 h period; Table S2. Data showing the First order, Zero order kinetic, Hopfenberg Model (Erosion Coefficient (m/h) and Ritger -Peppas model of SPEEK membrane SA02 with nalidixic acid sodium salt in 1 to 8 h period; Table S3. Data showing the First order, Zero order kinetic, Hopfenberg Model (Erosion Coefficient (m/h) and Ritger -Peppas model of SPEEK membrane SA01 with ciprofloxacin in 1 to 8 h period; Table S4. Data showing the First order, Zero order kinetic, Hopfenberg Model (Erosion Coefficient (m/h) and Ritger -Peppas model of SPEEK membrane SA02 with ciprofloxacin in 1 to 8 h period.
