# Supplementary material for: Design of an Antibiotic-Releasing Polymer: Physicochemical Characterization and Drug Release Patterns
Source: Membranes (Basel). 2023 Jan 12;13(1):102. doi: 10.3390/membranes13010102 (PMC9866011; doi:10.3390/membranes13010102)
Supplement: Supplementary file 1 [file membranes-13-00102-s001.zip › membranes-1978695-supplementary.pdf]

Supplementary Data

Design of an Antibiotic-Releasing Polymer: Physicochemical Characterization and Drug Release Patterns

Himabindu Padinjarathil [1,2,†], Srikrishna Mudradi [1,†], Rajalakshmi Balasubramanian [1], Carmelo Drago [3], Sandro Dattilo [4,*], Nikhil K. Kothurkar [2,5] and Prasanna Ramani [1,5*]

[1]Dhanvanthri Laboratory, Department of Sciences, Amrita School of Physical Sciences, Coimbatore 641112, Amrita Vishwa Vidyapeetham, India. p_himabindu@cb.students.amrita.edu (HB); cb.sc.p2che18015@cb.students.amrita.edu (SM); cb.sc.i5che16022@cb.students.amrita.edu (RB); r_prasanna1@cb.amrita.edu (PR).

[2]Department of Chemical Engineering and Materials Science, School of Engineering, Coimbatore 641112, Amrita Vishwa Vidyapeetham, India. k_nikhil@cb.amrita.edu (NKK)

[3]Institute of Biomolecular Chemistry, CNR, via Paolo Gaifami 18, 95126 Catania, Italy. carmelo.drago@cnr.it (CD)

[4]Institute for Polymer, Composite and Biomaterials, CNR, via Paolo Gaifami 18, 95126 Catania, Italy. sandro.dattilo@cnr.it (SD)

[5]Center of Excellence in Advanced Materials & Green Technologies (CoE–AMGT), Amrita School of Engineering, Coimbatore 641112, Amrita Vishwa Vidyapeetham, India

[*]Corresponding author(s): PR: r_prasanna1@cb.amrita.edu; SD: sandro.dattilo@cnr.it

[†]Contributed equally

Results

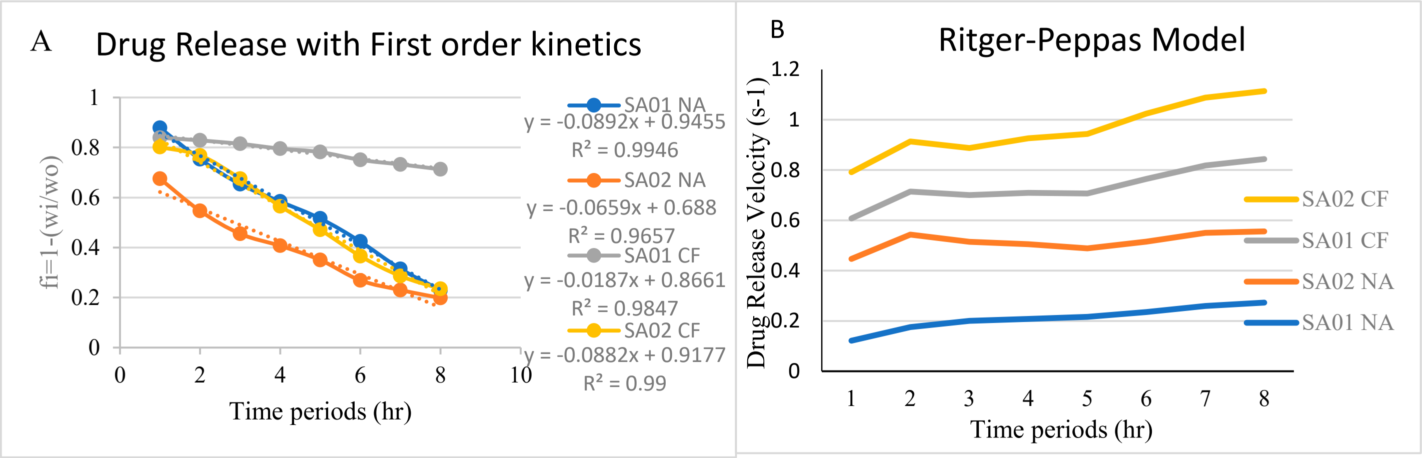

Figure S1. A) Graph illustration of linear equation of drug release from SPEEK membranes SA01 and SA02 with two different drug Nalidixic acid (NA) and ciproflaxcin (CF) to show first order kinetics. B) Graph illustration the Drug release velocity (n) from SPEEK membrane SA01 and SA02 with (Nalidixic Acid and Ciproflaxcin) in 1 to 8 hr period.

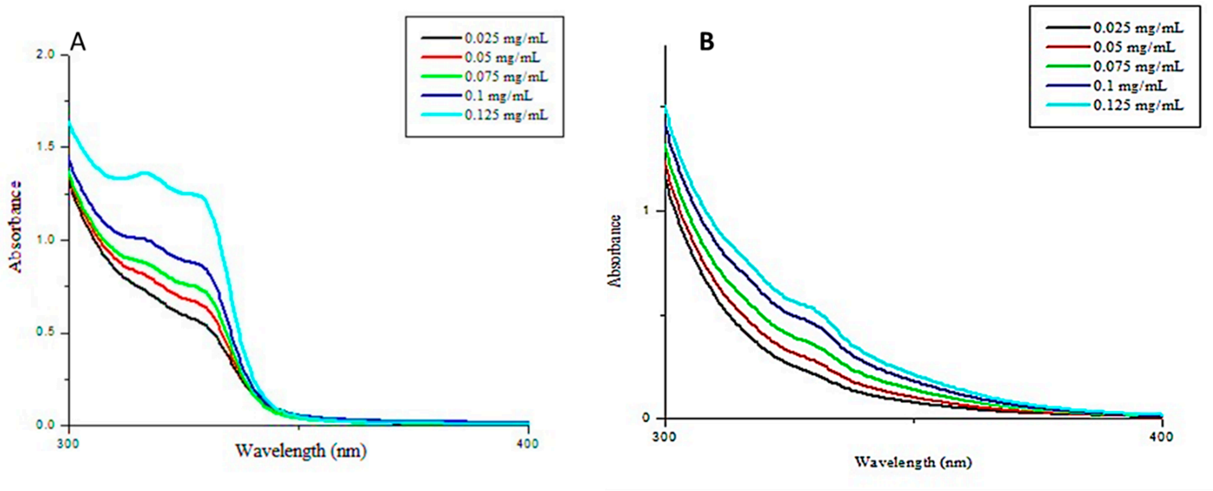

Figure S2. Drug in various concentraions A) Nalidixic acid (NA) and B) ciproflaxcin (CF).

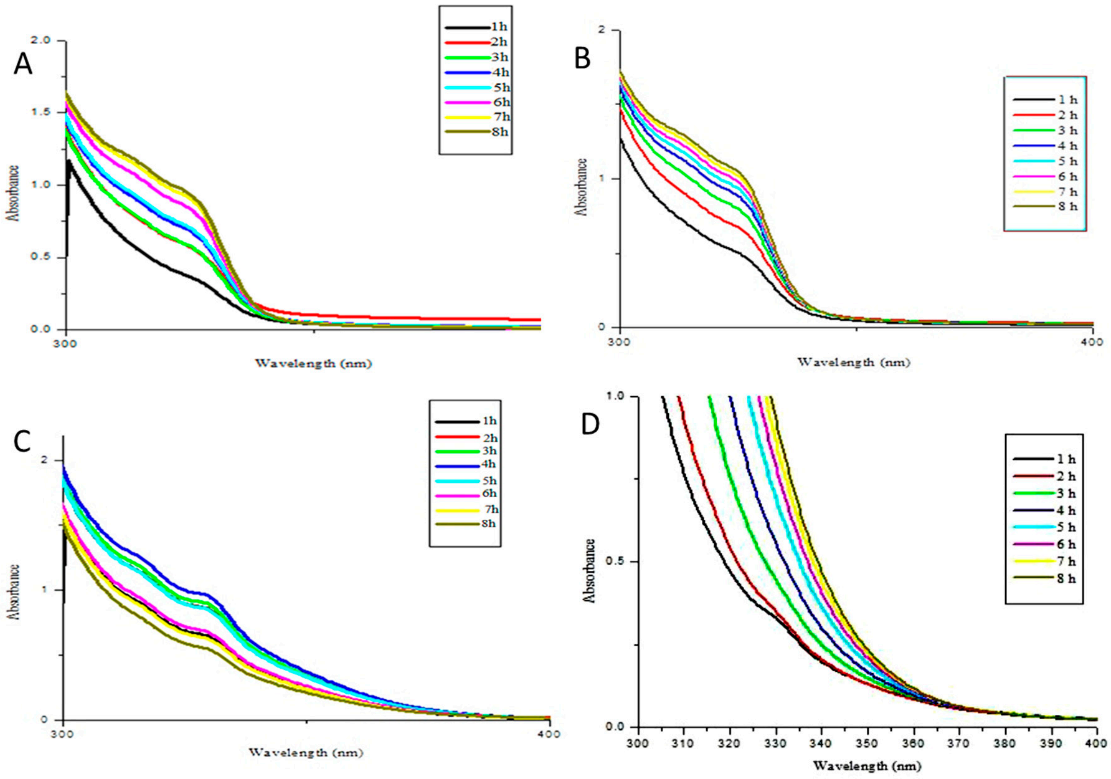

Figure S3: Linear equation of drug release from SPEEK membranes. A) SA01 and B) SA02with Nalidixic acid (NA). C) SA01 and D) SA02 with Ciprofloxacin for 1 to 8 hr period.

Table S1. Data showing the First order, Zero order kinetic, Hopfenberg Model (Erosion Coefficient (m/h) and Ritger -Peppas model of SPEEK membrane SA01 with nalidixic acid sodium salt in 1 to 8 hr period.

	**Hopfenberg Model**	**Ritger - Peppas Model**	**Zero Order Kinetics**	**First Order Kinetics**
Time (hr)	Erosion Coefficient (m/h)	Drug Release Velocity (s^{-1})	Rate Constant	Rate Constant (s^{-1})
1	5.22×10^{-4}	0.1215	0.1215	5.8562×10^{-4}
2	5.33×10^{-4}	0.1755	0.1755	1.936×10^{-4}
3	4.96×10^{-4}	0.2001	0.2001	9.811×10^{-5}
4	4.47×10^{-4}	0.2081	0.2081	6.089×10^{-5}
5	4.16×10^{-4}	0.2165	0.2165	4.0311×10^{-5}
6	4.12×10^{-4}	0.2352	0.2352	2.553×10^{-5}
7	4.2×10^{-4}	0.2591	0.2591	1.499×10^{-5}
8	4.14×10^{-4}	0.2725	0.2725	9.04×10^{-6}

Table S2. Data showing the First order, Zero order kinetic, Hopfenberg Model (Erosion Coefficient (m/h) and Ritger -Peppas model of SPEEK membrane SA02 with nalidixic acid sodium salt in 1 to 8 hr period.

	**Hopfenberg Model**	**Ritger - Peppas Model**	**Zero Order Kinetics**	**First Order Kinetics**
Time (hr)	Erosion Coefficient (m/h)	Drug Release Velocity (s^{-1})	Rate Constant	Rate Constant (s^{-1})
1	12.76×10^{-4}	0.325	0.325	3.1226×10^{-4}
2	10.21×10^{-4}	0.3677	0.3677	9.084×10^{-5}
3	7.13×10^{-4}	0.3147	0.3147	5.6211×10^{-5}
4	5.82×10^{-4}	0.2965	0.2965	3.63×10^{-5}
5	4.78×10^{-4}	0.2721	0.2721	2.7603×10^{-5}
6	4.82×10^{-4}	0.3013	0.3013	1.407×10^{-5}
7	4.32×10^{-4}	0.291	0.291	1.037×10^{-5}
8	3.94×10^{-4}	0.2835	0.2835	7.663×10^{-6}

Table S3. Data showing the First order, Zero order kinetic, Hopfenberg Model (Erosion Coefficient (m/h) and Ritger -Peppas model of SPEEK membrane SA01 with ciprofloxacin in 1 to 8 hr period.

	**Hopfenberg Model**	**Ritger - Peppas Model**	**Zero Order Kinetics**	**First Order Kinetics**
**Time (hr)**	Erosion Coefficient (m/h)	Drug Release Velocity (s^{-1})	Rate Constant	Rate Constant (s^{-1})
**1**	5.22 X 10^{-4}	0.1215	0.1215	5.8562 X 10^{-4}
**2**	5.33 X 10^{-4}	0.1755	0.1755	1.936 X 10^{-4}
**3**	4.96 X 10^{-4}	0.2001	0.2001	9.811 X 10^{-5}
**4**	4.47 X 10^{-4}	0.2081	0.2081	6.089 X 10^{-5}
**5**	4.16 X 10^{-4}	0.2165	0.2165	4.0311 X 10^{-5}
**6**	4.12 X 10^{-4}	0.2352	0.2352	2.553 X 10^{-5}
**7**	4.2 X 10^{-4}	0.2591	0.2591	1.499 X 10^{-5}
**8**	4.14 X 10^{-4}	0.2725	0.2725	9.04 X 10^{-6}

Table S4. Data showing the First order, Zero order kinetic, Hopfenberg Model (Erosion Coefficient (m/h) and Ritger -Peppas model of SPEEK membrane SA02 with ciprofloxacin in 1 to 8 hr period.

	**Hopfenberg Model**	**Ritger - Peppas Model**	**Zero Order Kinetics**	**First Order Kinetics**
**Time (hr)**	Erosion Coefficient (m/h)	Drug Release Velocity (s^{-1})	Rate Constant	Rate Constant (s^{-1})
**1**	7.63×10^{-4}	0.1983	2.227×10^{-4}	4.495×10^{-4}
**2**	4.45×10^{-4}	0.1638	1.067×10^{-4}	2.032×10^{-4}
**3**	4.16×10^{-4}	0.1873	6.256×10^{-5}	1.043×10^{-4}
**4**	4.18×10^{-4}	0.2175	3.924×10^{-5}	5.782×10^{-5}
**5**	4.07×10^{-4}	0.2365	2.616×10^{-5}	3.54×10^{-5}
**6**	4.065×10^{-4}	0.259	1.693×10^{-5}	2.1077×10^{-5}
**7**	3.92×10^{-4}	0.27	1.1337×10^{-5}	1.337×10^{-5}
**8**	3.68×10^{-4}	0.2706	8.148×10^{-6}	9.288×10^{-6}
